# Crucial Players for Inter-Organelle Communication: PI5P4Ks and Their Lipid Product PI-4,5-P_2_ Come to the Surface

**DOI:** 10.3389/fcell.2021.791758

**Published:** 2022-01-07

**Authors:** Archna Ravi, Lavinia Palamiuc, Brooke M. Emerling

**Affiliations:** Cell and Molecular Biology of Cancer Program, Sanford Burnham Prebys, La Jolla, CA, United States

**Keywords:** phosphoinositides, phosphotidylinositol-5-phosphate-4-kinases, PI-4, 5-P2, organelle, metabolism, peroxisomes, lipids

## Abstract

While organelles are individual compartments with specialized functions, it is becoming clear that organellar communication is essential for maintaining cellular homeostasis. This cooperation is carried out by various interactions taking place on the membranes of organelles. The membranes themselves contain a multitude of proteins and lipids that mediate these connections and one such class of molecules facilitating these relations are the phospholipids. There are several phospholipids, but the focus of this perspective is on a minor group called the phosphoinositides and specifically, phosphatidylinositol 4,5-bisphosphate (PI-4,5-P_2_). This phosphoinositide, on intracellular membranes, is largely generated by the non-canonical Type II PIPKs, namely, Phosphotidylinositol-5-phosphate-4-kinases (PI5P4Ks). These evolutionarily conserved enzymes are emerging as key stress response players in cells. Further, PI5P4Ks have been shown to modulate pathways by regulating organelle crosstalk, revealing roles in preserving metabolic homeostasis. Here we will attempt to summarize the functions of the PI5P4Ks and their product PI-4,5-P_2_ in facilitating inter-organelle communication and how they impact cellular health as well as their relevance to human diseases.

## Introduction

Lipids are essential components of cellular membranes. The type and the amount of lipids vary within the membranes at the organism, cell, as well as the organellar level. The lipid composition, quantitative and qualitative, of the membranes defines not only their physical characteristics but also, their biological properties. The majority of the membrane lipids are grouped into glycerophospholipids (GPLs), sphingolipids, and sterols (van Meer et al., 2008). GPLs are characterized by a glycerol backbone which forms the link between the head group that consists of a phosphate and an alcohol and the tail which consists of varying lengths of fatty acid chains (Harayama and Riezman, 2018). One such group of GPLs are the phosphoinositols (PIs).

PIs derive their name from the inositol head group, with the most predominant fatty acyl chains seen in the PIs in tissues being the stearoyl and arachidonoyl-acyl chains (Harayama and Riezman, 2018). PIs give rise to seven different species of phosphoinositides by the phosphorylation and dephosphorylation at the 3,4 and 5 position of the inositol moieties by the precise actions of specific kinases and phosphatases (Balla, 2013; Burke, 2018; Palamiuc et al., 2020). While found in minute amounts as compared to the other lipids on membranes, PIs play essential roles in regulating various cellular functions such as cytoskeletal remodeling, vesicular budding, membrane dynamics to regulating ion channels and signaling pathways. This is carried out by recruitment and interaction with, as well as activation of proteins, in a spatial and temporal manner (Falkenburger et al., 2010). Every species of phosphoinositide has its own cohort of interacting partners. This, along with the fact that membranes differ in their lipid composition, allows for distinct functional entities. In this review, we will mainly focus on one particular PI species, phosphatidylinositol-4,5-bisphosphate (PI-4,5-P_2_).

PI-4,5-P_2_, along with phosphotidylinositol-4-phosphate (PI-4-P), accounts for the bulk of all PIs (Di Paolo and De Camilli, 2006) and are generated by two distinct families of PI kinases. Initially these enzymes were identified as the Type I and Type II PI4P-kinases (Phosphatidylinositol-4-phosphate kinases) based on their biochemical properties and immunochemical cross-reactivities (Bazenet et al., 1990; Ling et al., 1989). It was later discovered that the Type II kinases actually produced PI-4,5-P_2_ by phosphorylating the 4-position of phosphatidylinositol 5-phosphate (PI-5-P), a previously unknown PI at time and interestingly the last PI to be discovered in higher organisms (Rameh et al., 1997), whereas the Type I kinases phosphorylated the 5-position of phosphatidylinositol 4-phosphate (PI-4-P) to produce the same PI-4,5-P_2_ species. Thus, giving rise to the current nomenclature of the PI families as the Type I kinases or PI4P5Ks (Phosphatidylinositol 4-phosphate 5-kinases) and the Type II or PI5P4Ks (Phosphatidylinositol 5-phosphate 4-kinases). Moreover, in mammalian cells, both kinase families have three distinct isoforms each, namely, α, β, and γ (Bulley et al., 2015; Clarke and Irvine, 2012; Clarke and Irvine, 2013). Apart from having very diverse immunological and catalytic functions, these kinases also generate PI-4,5-P_2_ on different cellular membranes (Table 1) (Balla, 2013).

**TABLE 1:** PI-4,5-P_2_ at the organelles: Binding partners and functions. PI-4,5-P2 interacting partners and organellar regulation. The table highlights some of the major interacting partners of PI-4,5-P_2_ on the plasma membrane, nucleus and other organelles and the functions they regulate. While the PM and nucleus functions are attributed to the type I kinases (PI4P5Ks), Golgi and ER have been shown to have roles regulated by PI-4,5-P_2_ generates by both family of kinases. Whereas autophagosome, lysosome and peroxisome interactions are carried out predominantly by PI-4,5-P_2_ generated by PI5P4Ks (Type II kinases).

Organelle	Binding partner(s)	Function	References
Plasma membrane	PLCδ	Hydrolysis to IP3 and DAG – second messengers	Falkenburger et al. (2013)
	CAPS, Synaptogamin1 and Syntaxin	Exocytosis	Martin (2015)
	AP2	Endocytosis	Jost et al. (1998)
	F-actin regulatory protein	Migration	Sun et al. (1999)
	?	Cell adhesion, spreading and migration	Yoneda et al. (2020)
	E-Syts on the ER	Ca^2+^ signaling	Giordano et al. (2013)
Nucleus	Pol I and Pol II	Transcription	Sobol et al. (2018)
			Sobol et al. (2013)
			Castano et al. (2019)
Endoplasmic reticulum	?	ER-Golgi transport	Itoh et al. (1998)
Golgi	Dynamin2, PAP1, PLD1	Transport carrier formation from TGN	Jones et al. (1998)
			Andreev et al. (1999)
			Freyberg et al. (2001)
	βIII-spectrin	Golgi-ER transport	Godi et al. (1998)
	ARNO family	Golgi structure	Monier et al. (1998)
Autophagosome	?	Inhibition of autophagy initiation	Vicinanza et al. (2015)
Lysosome	ESCRTIII (?)	Lysosome-autophagosome fusion and possibly cholesterol trafficking into the lysosome	Lundquist et al. (2018)
Peroxisome	Syt-7 on the lysosome	Trafficking of cholesterol from lysosome	Chu et al. (2015)
	E-Syts on the ER	Trafficking to cholesterol to the ER	Xiao et al. (2019)
	ESCRTIII (?)	Trafficking of VLCFA from LDs	Ravi et al. (2021)

### PI-4,5-P_2_ Functions on Cellular Membranes and Correlations for Organellar Cooperation

With PI-4-P being 10 times more abundant than PI-5-P on the plasma membrane (PM), PI-4,5-P_2_ generated here is predominantly by the action of Type I PIPKs or PI4P5Ks and constitute only 1–3% of the total lipid content on the membrane (King et al., 1987; King et al., 1989; McLaughlin et al., 2002). As a substrate, PI-4,5-P_2_ can be hydrolyzed to IP_3_ (inositol 1,4,5-trisphosphate) and DAG (diacylglycerol), by the activity of phospholipase C (PLC), which in turn serve as second messengers in various intracellular signaling cascades (Falkenburger et al., 2013). PI-4,5-P_2_ is also a precursor for PI-4-P and PI-3,4,5-P_3_ both of which play a role themselves in signaling and membrane dynamics (Auger et al., 1989; Varnai et al., 2006).

**FIGURE 1:**
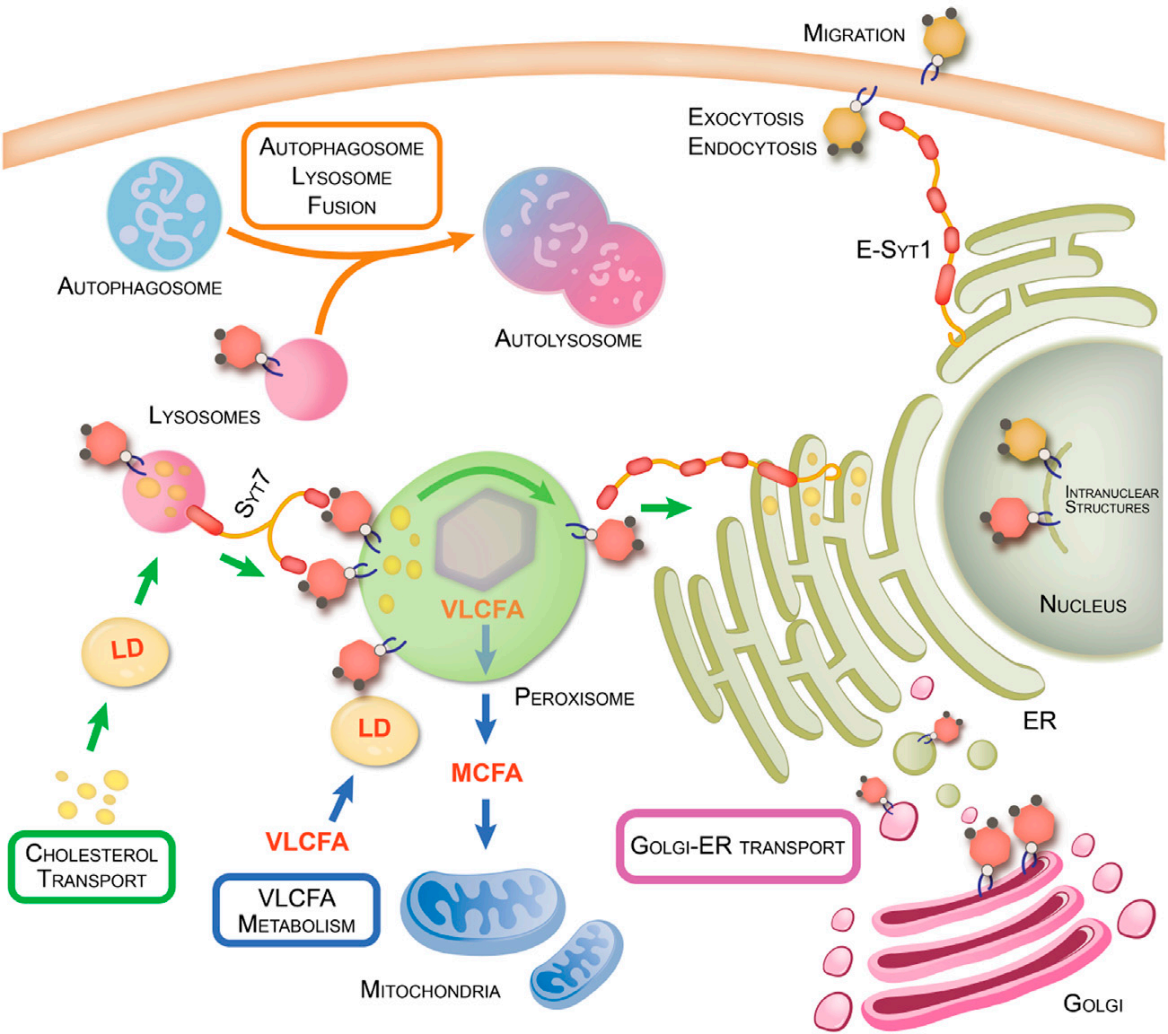
PI-4,5-P_2_ regulation of organellar interactions and cellular metabolism. Organelle-organelle interactions within the cell are key to regulating various cellular processes and transport of nutrients and materials to maintain metabolic homeostasis. Disruption of these interactions can lead to various diseased states such as cancer and neurodegenerative disorders. Here we summarize the various functions regulated by PI4P5K-generated PI-4,5-P_2_ (yellow hexagons) and PI5P4K-generated PI-4,5-P_2_ (red hexagons). This figure also highlights organellar interactions regulated by PI5P4Ks and their products as well the proteins involved and the metabolic pathways impacted by such interactions. Refer also to Table 1 for details of PI-4,5-P_2_ interactors and functions on the plasma membrane.

Additionally, PI-4,5-P_2_ plays a major role in membrane remodeling and trafficking. As will be discussed in the later sections, this function is essential not only at the level of the PM but is also a feature in key inter-organellar events mediated by these lipids to regulate cellular and metabolic homeostasis. At the PM, fusion and fission cycles can lead to exocytosis and endocytosis, both of which are invariably dependent on PI-4,5-P_2_. These are mediated by the ability of PI-4,5-P_2_ to bind various proteins via structured basic regions such as pleckstrin homology (PH) and C2-domains (Balla, 2013). PI-4,5-P_2_ is also a requisite for Ca^2+^ dependent PM-Endoplasmic Reticulum (ER) interaction by binding C2-domain containing E-Syts (Extended-synaptogamins) that are embedded in the ER membrane (Giordano et al., 2013). Interestingly, for a lipid that is quantitatively low on the PM, PI-4,5-P_2_ has shown to be a central mediator of a multitude of cellular functions and not surprisingly, loss of or mutation in kinases and phosphatases that regulate PI-4,5-P_2_ levels can lead to various diseased states (Pendaries et al., 2003; McCrea and De Camilli, 2009). Even though the bulk of the PI-4,5-P_2_ in the cell is generated by the Type I kinases, here we are focusing on the small but significant pool of PI-4,5-P_2_ that is created by the action of the Type II kinases, which to date is under appreciated yet is emerging to be fundamental for many essential cellular and metabolic events. The role of Type I kinases and their product is discussed in detail in another review (Katan and Cockcroft, 2020). Figure 1 and Table 1 also highlight some of the interactions mediated by PI-4,5-P_2_ generated via the activity of the Type I kinases.

With the Type I or PI4P5Ks being considered the main pathway for PI-4,5-P_2_ synthesis and PI-4-P being more abundant compared to the minor phosphoinositide, PI-5-P, the Type II or PI5P4Ks were relegated to the role of merely regulating the level of this lipid in the cells. However, recent studies have eloquently shown that PI-4,5-P_2_ generated by PI5P4Ks are not just by-products but have the capacity to modulate cellular metabolism by regulating organellar functions (Hu et al., 2018; Xiao et al., 2019; Ravi et al., 2021). Of the three isoforms described, PI5P4Kα is the most active and PI5P4Kγ the least active (Clarke and Irvine, 2013; Giudici et al., 2016). As a substrate, PI-5-P is also the most elusive of the phosphoinositides, with its separation and identification in the cell made difficult due to technical limitations. Since its discovery almost 25 years ago (Rameh et al., 1997), new roles and localization in the cellular compartments is being constantly uncovered. Studies have shown that the levels of PI-5-P change in response to various stimuli such as insulin, oxidative stress, bacterial infection etc. and in turn regulate numerous cellular functions such as cell signaling, vesicular transport and even play a role in the nucleus (Hasegawa et al., 2017; Ghosh et al., 2019).

One of the first biochemical studies of cellular compartments showed enrichment of PI-5-P at both the Golgi and the PM (Sarkes and Rameh, 2010). The product of its phosphorylation, PI-4,5-P_2_ has been detected on the stacked cisternae of the Golgi and its role has been studied in connection with maintaining the structural and functional integrity of the Golgi. PI-4,5-P_2_-mediated interactions with various proteins at the Golgi is essential for its structural organization, formation of carriers from the trans-Golgi network (TGN) as well as Golgi-ER transport (De Matteis et al., 2002; De Matteis and D'Angelo, 2007). Moreover, PI5P4Ks are functionally involved in various signaling events at the Golgi (Mackey et al., 2014). In fact, PI5P4Kγ has been localized to the ER suggesting PI-4,5-P_2_ synthesis at this organelle by these kinases (Itoh et al., 1998) and with PI-4,5-P_2_ playing a role in ER-Golgi transport, it remains to be seen whether and what percent of the lipid is generated by PI5P4Ks (De Matteis et al., 2002). However, this is contradictory to the studies demonstrating that the gamma isoform has very little inherent kinase function *in vitro* (Clarke et al., 2008). The mechanism by which PI5P4Kγ functions, and the role it plays in regulating cellular processes, remains to be explored further.

Interestingly, PI-5-P has also been found in the nucleus where it not only serves as a substrate for PI5P4Ks at that location but has been implicated in various nuclear outputs (Poli et al., 2019). PI5P4Kβ is the only isoform that has a unique nuclear localization signal that allows it to be targeted to the nucleus. However, the PI5P4Ks are dimeric enzymes and therefore the beta isoform has been shown to dimerize with the alpha, directing it into the nucleus as well (Bultsma et al., 2010). While PI-4,5-P_2_ has been shown to have nuclear functions such as the involvement with Pol I and Pol II mediated transcription as well as associating with splicing compartments, these functions have mostly been attributed to the canonical Type I PI4P5Ks. Thus far, the main function of the Type II PI5P4Ks at the nucleus seems to be quelling the starvation-induced increase in levels of PI-5-P. Details of the role of PI-4,5-P_2_ at the organelles mentioned thus far are discussed in Tan X et al. (2015) Also, see Table 1 for various interacting proteins of PI-4,5-P_2_ and the roles played in inter-organellar communication.

Vicinanza et al., show that PI-5-P is essential for autophagosome formation and overexpression studies showed that PI5P4Kγ localizes to the autophagosomes more frequently than both the PI5P4Kα and β isoforms. Knockdown of the kinases leads to the increase in the autophagosome formation, indicating that PI-4,5-P_2_ generation attenuates autophagosome biogenesis (Vicinanza et al., 2015). On the contrary another study showed that ATG16L1 interacts with PI-4,5-P_2_ on the PM to activate this process (Ravikumar et al., 2010). PI5P4Ks have also been shown to localize to the lysosome (Lundquist et al., 2018). Further, the loss of PI5P4Kα and PI5P4Kβ is sufficient to prevent the fusion of autophagosomes with lysosomes, which inhibits the process of autophagy and leads to accumulation of autophagosomes. This function of the PI5P4Ks requires a concomitant loss of p53 or a similar cellular stress (Lundquist et al., 2018). Together, these data indicate an important role for PI-4,5-P_2_ in the process of autolysosome formation. Moreover, a recent study showed that the endosomal sorting complex required for transport (ESCRT) is essential for lysosomal membrane repair (Gupta et al., 2021). These complexes are involved in membrane remodeling, which follows fusion events such as those between organelles. Interestingly, ESCRT complex proteins interact specifically with PI-4,5-P_2_ (McCullough et al., 2015), which could explain the importance of the lipid on the lysosomal membrane and its role in autophagy. For an in-depth discussion of PI-4,5-P_2_, and other phosphoinositide in autophagy, refer to Palamiuc et al. (2020).

Studies have also shown the localization of PI5P4Kα and the role of the PI5P4Ks in general at the peroxisomes. Peroxisomes, though highly essential for regulating various metabolic functions, are not well studied in mammalian cells. In the recent years, peroxisomes, their actions, as well as their role in regulating lipid metabolism have come to the forefront, bringing with them the PI5P4Ks and their product, PI-4,5-P_2_ into the spotlight.

### PI5P4Ks as Key Regulators of Peroxisomal Functions by Sustaining PI-4,5-P_2_ Homeostasis

Peroxisomes are single-membraned organelles with multifaceted functions, ranging from ether lipid biosynthesis to fatty acid (FA) oxidation and reactive oxygen species (ROS) metabolism (He et al., 2021). They respond to metabolic and environmental cues, putting them at the center of various signaling nodes in the cell. Also, because of their roles, peroxisomes interact with and regulate other organellar functions such as the lysosomes, lipid droplets (LDs) and the mitochondria (He et al., 2021). Multiple studies have placed PI-4,5-P_2_ at the peroxisomes (Jeynov et al., 2006; Chu et al., 2015; Ravi et al., 2021). Furthermore, recent work has showed that this pool of PI-4,5-P_2_ is generated by PI5P4Kα (Hu et al., 2018). We, in a recent publication, were the first to physically localize PI5P4Kα to the peroxisome in the mouse prostate tissue (Ravi et al., 2021). Further, using imaging techniques, we showed the localization of PI-4,5-P_2_ to the peroxisomes, confirming the lipid blot data from Chu et al. We also categorically demonstrated that the knockout of the two most active isoforms of the Type II kinases, namely, PI5P4Kα and PI5P4Kβ were sufficient to deplete the peroxisomal pool of PI-4,5-P_2_ and this can be rescued by adding back the wild-type (WT) but not by the kinase-dead PI5P4Kα (Ravi et al., 2021).

PI-4,5-P_2_, as mentioned earlier, can bind to a wide range of proteins with PH- or C2- domains to mediate diverse cellular functions. Similarly, as shown by Chu et al., peroxisomal PI-4,5-P_2_ interacts with the C2 domain containing Synaptogamin VII (Syt7) on the lysosomal membrane to regulate cholesterol trafficking from the lysosome to the peroxisome. Their work revealed a previously unappreciated role for peroxisomes in cholesterol transport. While NPC1 (Niemann-Pick disease, type C1) on lysosomes and ABCD1 (ATP Binding Cassette Subfamily D Member 1) on peroxisomes are indispensable for the formation of lysosome-peroxisome membrane contact (LPMC), Syt7 allows for the stabilization of these sites by binding to PI-4,5-P_2_ on the peroxisome. Knockdown on Syt7 leads to accumulation of cholesterol in the lysosomes of the cells, uncovering a crucial role for the LPMC in cholesterol transport (Chu et al., 2015). In their following paper, they further demonstrated that knockdown of PI5P4Kα but not PI5P4Kβ or PI5P4Kγ leads to accumulation cholesterol in the lysosome, similar to the loss of Syt7 (Hu et al., 2018). Notably, we showed that the β isoform of the kinases is also able to rescue, albeit to a lesser extent than the α isoform, PI-4,5-P_2_ on peroxisomal membranes (Ravi et al., 2021). This correlates with the fact that PI5P4Kα is the most catalytically active of the isoforms and also may explain why Hu et al., did not observe a marked increase in cholesterol accumulation in the lysosome upon PI5P4Kβ or PI5P4Kγ knockdown. They also go on to show that while the loss of PI5P4Kα is sufficient to regulate peroxisomal PI-4,5-P_2_ it does not seem to affect lysosomal PI-4,5-P_2_ (Hu et al., 2018). This supports the role of the PI5P4Ks in autophagosome-lysosome fusion, which requires the loss of both the α and the β isoforms (Lundquist et al., 2018). Since both lysosomes and peroxisomes are sites of localization for the PI5P4Ks, it was also elegantly demonstrated *in vitro* that, peroxisomes and not lysosomes isolated from PI5P4Kα knockdown affect LPMC formation, conclusively showing the role of PI-4,5-P_2_ on the peroxisomes in this process (Chu et al., 2015). Further, similar to the PM-ER interaction, the PI-4,5-P_2_ on the peroxisomes also binds E-Syts on the ER, to traffic cholesterol. This lysosome-peroxisome-ER transport explains a previously not understood mechanism about the how exogenous cholesterol is trafficked from lysosomes to the ER (Xiao et al., 2019).

Other than cholesterol metabolism, peroxisomes are also sites of breakdown of very long chain fatty acids (VLCFAs) to medium chain fatty acids (MCFAs), known as peroxisomal β-oxidation (Poirier et al., 2006). Our study has shown a role for the PI5P4Ks in peroxisomal β-oxidation as seen by the change in peroxisomal gene signature upon loss of α and β isoforms (Ravi et al., 2021). For this process to occur, the peroxisomes need to take up VLCFAs, which are stored in the form of LDs. Concisely, peroxisome-LD interactions, which as with the LPMC formation, require ABCD1 on peroxisomal membranes and M1 Spastin on LDs, following which the FAs are trafficked across the membranes of the organelles (Chang et al., 2019). While loss of the PI5P4Ks does not affect the peroxisome’s interaction with LDs, they are no longer able to take up the FAs. This phenotype can be rescued by adding back the WT but not the kinase-dead PI5P4Kα, indicating that PI-4,5-P_2_ is essential for the trafficking event (Ravi et al., 2021). Chang et al., showed that upon tethering LDs to the peroxisomes, M1 Spastin is also responsible for recruiting ESCRT-III proteins to the surface, which is essential for FA uptake (Chang et al., 2019). Interestingly, as previously mentioned, ESCRT-III complex proteins preferentially associate with PI-4,5-P_2_. This could provide a possible explanation for the involvement of PI-4,5-P_2_ in regulating FA trafficking. The chain shortened FAs from peroxisomal β-oxidation are then utilized by mitochondria to break them down into carbon dioxide and water, in a process that generates ATP (Fransen et al., 2017). Studies have shown that peroxisomal storage and biogenesis disorders lead to mitochondrial dysfunction (Lismont et al., 2015; Tanaka et al., 2019). Similarly, knockdown of the PI5P4Ks leads to major structural and functional defects in the mitochondria downstream of peroxisome dysregulation (Ravi et al., 2021). These two organelles not only play a key role in lipid metabolism, but in redox mechanisms as well, tying them together in maintaining cellular homeostasis and PI-4,5-P_2_ generated by the PI5P4Ks is surfacing to be crucial in maintaining this balance. See Figure 1 and Table 1.

### Health and Disease: PI5P4Ks Emerge as Exciting Targets

It is not surprising, considering the wide-ranging role PI-4,5-P_2_ plays, that PI5P4Ks will have such a drastic effect on cellular signaling and metabolism. Depending on the disease state, the activity of PI5P4Ks can be leveraged to bring about a change in the system. While targeting a kinase might seem like a daunting task, PI5P4Ks are a more feasible target. This is predominantly because their substrate, PI-5-P, mainly accumulates under conditions of stress. Our previous work demonstrated that PI5P4Ks are essential for tumor formation upon loss of p53, suggesting the PI5P4Ks are attractive targets for p53 mutant cancers (Emerling et al., 2013) and become relevant in regulating autophagosome-lysosome fusion under these conditions (Lundquist et al., 2018). Further, we have shown elevated expression of the PI5P4Ks in breast tumors compared to normal breast tissue (Emerling et al., 2013) and other studies have shown the impact of the kinases in breast cancer (Luoh et al., 2004; Keune et al., 2013). Similarly, PI5P4Ks have been demonstrated to be upregulated in several cancer subtypes, including glioblastomas, AML, and sarcomas (Fiume et al., 2015; Jude et al., 2015; Zhang et al., 2018; Lima et al., 2019; Shin et al., 2019; Ravi et al., 2021). Moreover, we recently illustrated the requirement of the PI5P4Ks for not only the establishment of sarcoma tumors but also in maintaining them, possibly through their role in regulating peroxisome-mitochondrial interplay (Ravi et al., 2021). While in diseases such as cancer, it might be necessary to inhibit the PI5P4Ks, other diseases/conditions might benefit from enhancing their activity. Accordingly, inhibiting autophagy is an appealing strategy in cancer therapeutics, whereas the reverse is true from an ageing standpoint. Studies have shown that PI-4,5-P_2_ levels are often lowered in neurodegenerative disorders, such as Alzheimer’s disease (Arancio, 2008). Lysosomal and peroxisomal storage disorders, encompass a large group of metabolic diseases, which are associated with the inability of the cells to properly process their cargo, such as cholesterol. These could serve as prime targets where we could possibly exploit the enzymatic function of the PI5P4Ks to our advantage. All things considered, in the recent past, PI5P4Ks and their product PI-4,5-P_2_ have risen from insignificance to being without a doubt one of the key metabolic sensors and regulators within the cell as well as pivotal players for inter-organelle communication necessary for cell survival. With drugs being developed against these kinases (Davis et al., 2013; Clarke et al., 2015; Al-Ramahi et al., 2017; Kitagawa et al., 2017; Manz et al., 2020; Sivakumaren et al., 2020; Chen et al., 2021), targeting them in the near future in various diseases is looking brighter.

## Data Availability

The original contributions presented in the study are included in the article/supplementary material, further inquiries can be directed to the corresponding author.
